# The Evolution of a Professional Practice Forum: Balancing Peer-to-Peer Learning With Course Objectives

**DOI:** 10.2196/resprot.3287

**Published:** 2014-10-31

**Authors:** Anna Janssen, Tracy Robinson, Tim Shaw

**Affiliations:** ^1^Workforce Education and Development GroupSydney Medical SchoolUniversity of SydneySydneyAustralia

**Keywords:** medical education, peer-to-peer, online learning, formative assessment

## Abstract

**Background:**

The Opioid Treatment Accreditation Course (OTAC) is a mandatory accreditation requirement in New South Wales, Australia, and aims to prepare medical practitioners for the provision of safe and effective Opioid Substitution Treatment to people with opioid dependence. The course has a strong focus on safe prescribing practices and the course design includes a Professional Practice Forum that is engaging for participants and effective at imparting complex ideas and concepts that do not place additional time constraints on already time-poor health professionals.

**Objective:**

The study aimed to use participatory action research methods to develop and evaluate an online Professional Practice Forum that is a key component of the OTAC teaching and learning experience.

**Methods:**

Three evaluation cycles were implemented with three cohorts of participants (N=40) to inform the design and review of the updated OTAC course. Overall, the study relied on participatory action research methods to enhance a sense of online community and to revise the Professional Practice Forum component of the course. Findings from survey feedback and an examination of Web metrics were used to monitor participant learning and were subsequently subject to thematic analysis in order to identify key themes.

**Results:**

The use of participatory action techniques in the redesign of the OTAC course was a successful means of engaging with participants and resulted in four revisions based on feedback from facilitators and participants. The Professional Practice Forum was rated highly and received positive feedback from both moderators and participants.

**Conclusions:**

The use of interactive forums in online learning in an educational module for adult learners can prove extremely valuable as a means for participants to share their expertise and improve their learning outcomes. In particular, the use of sticky and welcome threads were significant features that enhanced interactions between participants and facilitators and resulted in increased quantity and quality of postings. These findings can help inform future researchers on how to develop peer engagement modules that are amenable to assessment and that build an online sense of community.

## Introduction

### Overview

Over the last decade, there has been increasing demand for continuing medical education programs that enhance the clinical skills and knowledge of medical specialists and general practitioners [1,2]. This is particularly the case in relation to providing safe treatment for people with opioid dependence because of the widespread stigma associated with opioid use in Australia. At the same time, medical practitioners have high-volume work environments and are often time-poor. Hence, educators need to develop high-quality learning environments and to identify and evaluate learning outcomes to ensure they have utility for participants and can be readily applied into their own practice [3,4]. The current Opioid Treatment Accreditation Course (OTAC) is designed around the core principles of both adult learning and situated learning [5-7]. This approach recognizes the knowledge and experience that participants bring to learning environments, and the literature confirms that this approach contributes to the creation of authentic and “real life” activities and contexts to promote learning [8].

The use of online forums and peer-to-peer learning approaches are now commonplace in e-learning because of their capacity to engage participants and to promote interactive learning environments [9,10]. In addition, the literature confirms that peer-to-peer learning approaches are particularly appealing in online medical education because they emphasize individual autonomy and participants are able to take responsibility for their own learning experiences [11]. Furthermore, collaborative educational environments allow adult learners to identify their existing knowledge and determine their future educational needs [12].

Nevertheless, the literature identifies high rates of attrition from online courses and that educators need to ensure the online environment is engaging and promotes a sense of community between participants [13]. Although challenging, a systematic review of 57 studies on e-learning for health professionals and students demonstrated that well-designed e-learning packages are learner centric and share responsibility between trainers and learners [14]. A significant challenge to building a thriving online community is the time and effort required to build a sense of community that requires enabling efforts more than moderation of participant activity [15]. This paper describes the development and review of an online course and Professional Practice Forum for clinicians who wish to be accredited to dispense Opioid Substitution Treatment in New South Wales (NSW), Australia.

### Background to the Opioid Treatment Accreditation Course

The treatment of opioid dependence is often challenging for medical practitioners and prescribing opioids in NSW occurs within a regulatory framework that requires clinicians to have approval from the NSW Health Department to prescribe to each patient [16]. The OTAC can be completed as either an online course or as a 1-day face-to-face workshop. In 2009, addiction medicine was formally recognized by the Australian government as a medical specialty. Given the complex mental and physical health needs experienced by people recovering from opioid dependence, it is anticipated that this recognition will improve the safety and standards of health care for this cohort. It is noteworthy, however, that not all practitioners who complete the OTAC course go on to be active prescribers.

The OTAC course primarily targets general practitioners, who are already heavily targeted for continuing medical education, much of which is online. Hence, medical practitioners have high expectations in relation to the quality of online materials and are well placed as informants for the design and application of online learning programs [2]. In 2011, the Workforce Education and Development Group at the University of Sydney undertook a content and design review of the OTAC and subsequently piloted the program with medical practitioners (N=14) from diverse locations, including regional and rural centers. The aim of the review was to realign the course with the latest approaches in online learning in the medical sector and to ensure that the program was consistent with the principles of adult education that include self-directed learning and knowledge acquisition [14,15]. Subsequently, the OTAC was implemented and evaluated with two more cohorts to further develop and refine the course components and, in particular, the Professional Practice Forum.

Providing participants with opportunities to consult experts and collaborate with their peers was seen as instrumental in exploring their attitudes to prescribing and in developing support and knowledge networks that could be sustained subsequent to their participation in the course. Hence, the updated OTAC consisted of three modules that allowed participants to be self-directed in attaining their learning objectives over the 4-week course duration and a Professional Practice Forum (the third module) that was a mandatory component for satisfactory course completion. This forum was designed to enable participants to be assessed by experienced prescribers (referred to as facilitators) to ensure they had achieved key learning outcomes and to allow participants an opportunity to reflect on and demonstrate the knowledge they had acquired.

The forum provided a platform for participants to collaborate and to reflect on their learning in an online community environment. Two experienced facilitators were recruited to moderate the forum and to formatively assess the extent to which participants had developed their knowledge and skills in line with the core learning principles of the wider course. Given the challenges involved in recruiting busy practitioners for lengthy periods of time, participants were only required to interact with peers and moderators for a maximum of 4 hours. The forum was a largely asynchronous activity, and this allowed participants to access the discussion at a time of their convenience. The forum was made available for a 4-week period and participants had a 5-day period in which they could interact with, and be assessed by, the moderators.

This paper reports on the modification and evaluation of the Professional Practice Forum designed to increase the knowledge and confidence of medical practitioners to become accredited prescribers in the OTAC program. In addition to reviewing and redesigning the course, the study used participatory action research (PAR) approaches in order to identify the salient factors for sustaining an online collaborative network in the field of opioid substitution. The study hypothesized that the use of PAR to design the Professional Practice Forum would enhance the learning experience and retention of participants in the course. PAR has been described as a collective and self-reflective inquiry that researchers and participants undertake in order to understand and improve on the practices in which they participate and the situations in which they find themselves [17]. In the current study, PAR methods were used to inform the development and refinement of the Professional Practice Forum.

## Methods

The current study involved three evaluation cycles of the OTAC with three cohorts of participants (N=40) and two facilitators who were also accredited and experienced prescribers. Participants were recruited primarily via email mail-out of course flyers, but some promotion was done via phone. Process and impact evaluations were undertaken after each of the three course iterations. In particular, observational techniques were used to assess processes such as peer interaction and the use of online resources. Analysis of website metrics was also undertaken, including the time that elapsed between the commencement of the forum module and when participants posted their first response. Interactions on the Professional Practice Forum were observed and recorded by the course coordinator in order to review the extent of interaction between participants and between participants and facilitators. Outcome measures were assessed using self-report surveys that asked participants to rate their learning experiences and to comment on their online sense of community. Permission to conduct this study was received from the University of Sydney’s Human Research and Ethics Committee.

The Professional Practice Forum was piloted during 2011. At the completion of the first two modules, all participants were emailed by the course coordinator and invited to submit a short scenario to the forum that demonstrated their understanding and application of the previous course modules. In addition, participants were asked to comment on the scenarios of at least 2 peers in order to foster engagement and team problem solving. Subsequent to their completion of the OTAC, participants were asked to respond to an evaluation on their experiences with the forum via an online survey. The survey measured their interest in the Professional Practice Forum format as well as the ease of use and any changes they would recommend. In addition, the forum facilitators were asked to provide feedback on the challenges and benefits of the forum in relation to its utility and how effective it was for enhancing participant knowledge and confidence.

The findings from the pilot study informed revisions to the Professional Practice Forum and the revised OTAC was subsequently implemented with (N=13) participants. This iteration of the course included four “sticky” threads that linked to exemplars of case scenarios, and participants were invited to respond to at least one scenario prior to submitting their own case studies. All other evaluation methods were consistent with the pilot study. A final iteration of the OTAC course was developed and tested in mid-2011 (N=13) This version of the course included a welcome thread that provided participants with an opportunity to access information on the backgrounds and expertise of course facilitators and to post their own biographical information. In addition, a feedback thread was included to enable an accessible and ongoing evaluation tool for future OTAC participants.

**Figure 1:**
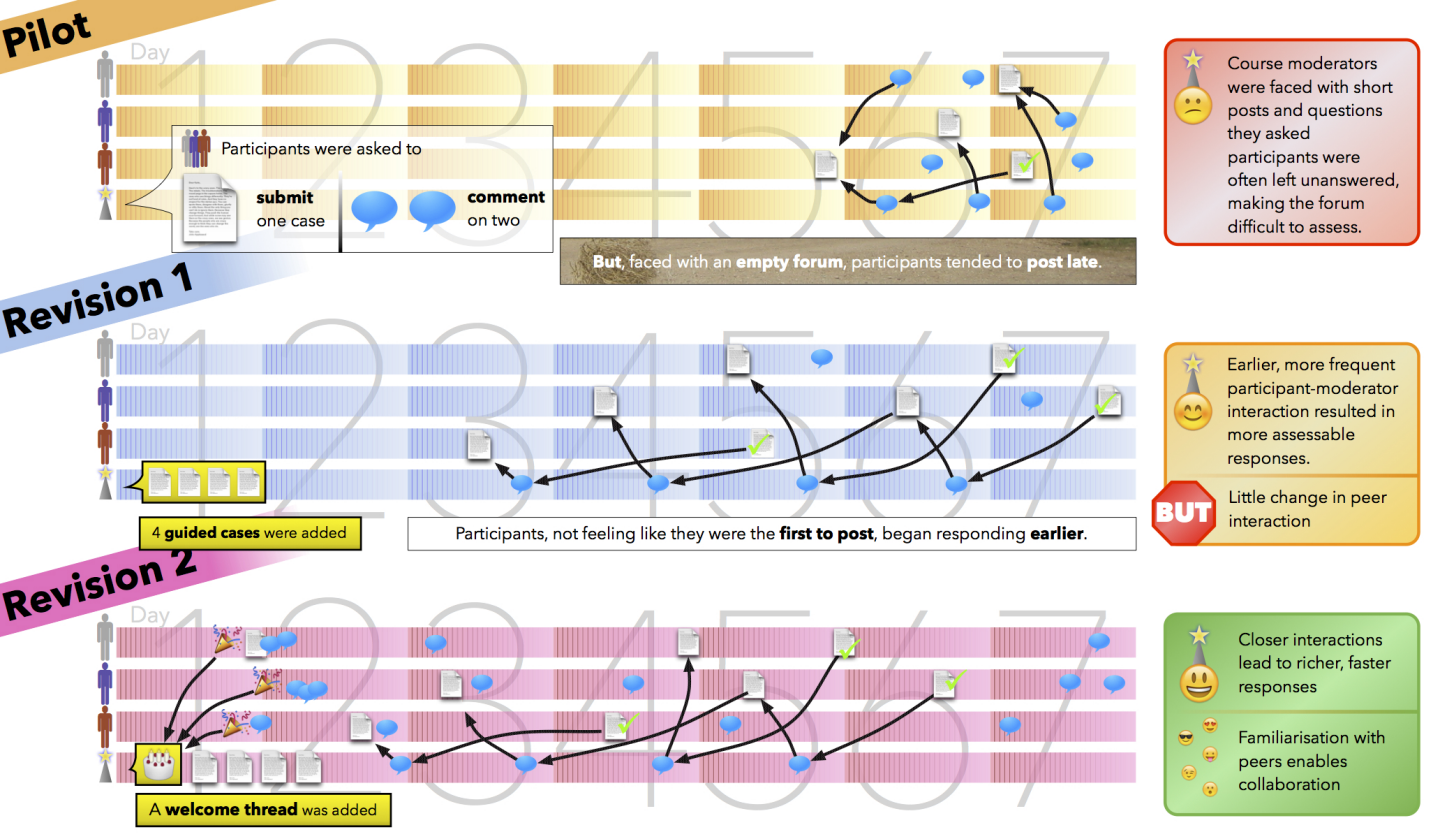
Visualization of the three evaluation phases, which resulted in revisions to improve the educational impact of the Professional Practice Forum.

Overall, the study used PAR methods to identify strategies for creating a sense of community and to revise the Professional Practice Forum. Feedback from each of the three course iterations was subject to thematic analysis that was undertaken until saturation was reached and clear themes emerged. See Figure 1.

## Results

A total of 40 participants completed all aspects of the OTAC and provided process and impact feedback. Course participants included 14 general practitioners, 6 registrars, 1 psychiatrist, and 2 chief medical officers; 15 participants indicated they were from rural and regional NSW and the remainder were based in urban centers. Findings that emerged from the feedback (surveys and Web metrics) demonstrate that the use of participatory action techniques in the re-design of the OTAC program was a successful means of engaging with participants and resulted in four revisions based on feedback from both facilitators and participants.

In the pilot OTAC, the open-interfaced design of the Professional Practice Forum appeared to discourage participants from making initial posts, which meant the course coordinator had to repeatedly email instructions and reminders to participants. The inclusion of four “sticky” threads in the second iteration of the course reduced the time participants took between commencing the course and posting their first discussion thread. It also allowed more discussion time for moderators and participants and a longer timeframe for peer interaction. In addition, the guidance provided by the sticky cases resulted in more pertinent responses and content between participants as well as lengthier posts that contained more detailed information about treatment options and management.

Feedback from facilitators’ observations of the Professional Practice forum indicated that some participants remained hesitant about fully engaging with their peers. This resulted in a third iteration of the OTAC that included a welcome thread. Participants reported that this had a notable impact on their engagement and sense of online community, which was demonstrated by their increased number of posts as well as the frequency and depth of their interactions with each other. The welcome thread allowed participants to introduce themselves and build rapport with their peers. As one participant stated, “I enjoyed this component and it was interesting to see the various challenges that all clinicians have at their point of treatment or engagement”.

Overall, participants reported that they enjoyed the OTAC course and that it resulted in improved knowledge about prescribing and managing people with opioid dependence. In particular, they enjoyed the opportunity of engaging with facilitators who had extensive experience in the field. As one participant stated, “It is an excellent teaching session, I learned a lot from seniors”. Nevertheless, facilitators did report that their engagement with and observations of the Professional Practice Forum were time consuming and that some participants required considerable encouragement to complete all forum activities. The use of PAR methods resulted in three changes to the OTAC and provided important information on the factors that enabled participation in the forum.

## Discussion

### Principal Findings

The re-design of the OTAC Professional Practice Forum was informed by literature on autonomous learning [1,2] and collaborative peer learning [11,14]. Most participants were able to commit approximately 4 hours of their time over the 7-day period to engage with forum activities. Nevertheless, facilitators and the course coordinator reported that they had to be proactive to ensure that all participants engaged fully with the course. This highlights the challenges involved in creating an evolving online community rather than choreographing participation. It also highlights the important role of “enabling” rather than moderating online facilitation. The use of current prescribers as facilitators was an important strategy for engaging participants and for enabling them to actively join the forum.

At the same time, facilitators reported challenges in facilitating the forum, particularly in relation to the administration burden of following up with participants in the first two iterations of the OTAC. Previously, participants had a tendency to post only what was required and to post directly to facilitators, without drawing on the expertise of their peers. The fact that participants increased their engagement with each other in the final OTAC demonstrates that when they are able to familiarize themselves with each other and when they are provided with exemplars of the work that is required, they are much more likely to actively contribute to the forum.

Another challenge with the creation of online forums is ensuring that participants post enough detail in their responses to make them amenable to both formative and summative assessment processes. The incorporation of the four sticky cases into the Professional Practice Forum after the initial pilot was an effective tool for encouraging participants to post comments on the forum with sufficiently detailed responses. This allowed facilitators to more accurately assess participant knowledge and meant there was more time for them to interact with participants and to request additional information. In the first iteration of the OTAC, participants were disinclined to post to the forum and this challenge is consistent with the literature that identifies participant engagement as a significant challenge in online learning [9].

In subsequent revisions of the OTAC that used guided and exemplar cases, participants were observed to make earlier postings and provided more detailed answers, which were noticeably longer and contained more detailed information on patient history and other diagnostic criteria. This demonstrated their depth of knowledge in regard to symptoms, treatment planning, and management. In the final revision of the Professional Practice Forum, the inclusion of a welcome thread had a noticeable impact on peer interaction. The use of the welcome thread provided participants with immediate access to the Professional Practice Forum and enhanced the growth of an online community. This highlights the importance of encouraging early online interaction between participants and recognizing that learning is also a social process. This is consistent with learner- and community-centered approaches to teaching and learning that emphasize the importance of building on participant knowledge, providing and receiving feedback, and self-evaluation.

### Limitations

Several limitations to the current study must be acknowledged. The use of purposive sampling and the diverse discipline backgrounds of participants means that the findings are not generalizable and further dissemination and evaluation of the OTAC course is warranted. In particular, future evaluations of the course should include follow-up information on whether participants go on to become active prescribers. At the same time, the sample was sufficient for the development and piloting of the Professional Practice Forum. The reliance on qualitative feedback from participants is another limitation, and the use of validated measures for online sense of community would be beneficial for quantifying the sense of connection between and within participants and facilitators. Furthermore, future evaluations of OTAC should include long-term follow-up with participants to ascertain if changes in their knowledge are sustained.

The use of PAR methods did, however, provide rich feedback on modifications that were required of the OTAC and resulted in an increased number and quality of interactions in the Professional Practice Forum. Given that the study aimed to build and sustain an online learning community and to enhance retention of busy clinicians, the methods used were adequate for the pilot implementation and refinement of the OTAC.

### Conclusions

The challenges of developing and sustaining a sense of community in online learning environments are well documented in the literature. However the literature has a particular focus on strategies for enhancing the sense of connection between participants and facilitators. There is still limited research on how to generate peer-to-peer interactions and harness them as a vehicle for developing and implementing online forums. The findings from this study contribute to better understanding of the factors that encourage peer-to-peer learning.

Peer-to-peer engagement in an educational module for adult learners can prove extremely valuable as a means for participants to share their expertise and improve their learning outcomes. The use of a forum module allowed the course designers to use both formative and summative assessment and evaluation processes. Given the relatively small number of participants, however, it is important that the OTAC is tested with a larger cohort of participants to further explore its utility across different geographical contexts where the use of online education may be less familiar. The use of participatory action approaches in the conduct of the current study was highly effective for allowing participants to engage actively in the construction of the Professional Practice Forum.

## Abbreviations

NSW: New South Wales
OTAC: Opioid Treatment Accreditation Course
PAR: participatory action research
